# Differential recognition patterns of *Schistosoma haematobium* adult worm antigens by the human antibodies IgA, IgE, IgG1 and IgG4

**DOI:** 10.1111/j.1365-3024.2010.01270.x

**Published:** 2011-03

**Authors:** F MUTAPI, C BOURKE, Y HARCUS, N MIDZI, T MDULUZA, C M TURNER, R BURCHMORE, R M MAIZELS

**Affiliations:** 1 Ashworth Laboratories, Institute of Immunology & Infection Research, School of Biological Sciences, University of EdinburghEdinburgh, UK; 2 National Institute of Health ResearchHarare, Zimbabwe; 3 Department of Biochemistry, University of ZimbabweHarare, Zimbabwe; 4 Glasgow Biomedical Research Centre, Faculty of Biomedical and Life Sciences, University of GlasgowGlasgow, UK; 5 Division of Infection and Immunity, Institute of Biomedical Life Sciences, University of GlasgowGlasgow, UK

**Keywords:** isotype, proteomics, recognition patterns, schistosomiasis

## Abstract

*Schistosoma haematobium* antigen recognition profiles of the human isotypes IgA, IgE, IgG1 and IgG4 were compared by image analysis of western blots. Adult worm antigens separated by two-dimensional gel electrophoresis were probed with pooled sera from Zimbabweans resident in a *S. haematobium* endemic area, followed by the identification of individual antigenic parasite proteins using mass spectrometry. Overall, IgG1 reacted with the largest number of antigens, followed by IgE and IgA which detected the same number, while IgG4 detected the fewest antigens. IgE recognized all antigens reactive with IgG4 as well as an additional four antigens, an isoform of 28-kDa GST, phosphoglycerate kinase, actin 1 and calreticulin. IgG1 additionally recognized fatty acid–binding protein, triose-phosphate isomerase and heat shock protein 70, which were not recognized by IgA. Recognition patterns varied between some isoforms, e.g. the two fructose 1-6-bis-phosphate aldolase isoforms were differentially recognized by IgA and IgG1. Although the majority of *S. haematobium* adult worm antigens are recognized by all of the four isotypes, there are clear restrictions in antibody recognition for some antigens. This may partly explain differences observed in isotype dynamics at a population level. Differential recognition patterns for some isoforms indicated in the study have potential importance for vaccine development.

## Introduction

Schistosomiasis is a major human parasitic disease in tropical and subtropical countries in Africa, the Middle East and South America (1). Urinary schistosomiasis caused by *Schistosoma haematobium* affects over 100 million people, and a recent survey in sub-Saharan Africa indicated that 70 million individuals had experienced haematuria and 32 million dysuria associated with *S. haematobium* infection (http://www.who.int/vaccine_research/diseases/soa_parasitic/en/index5.html Accessed 16th September 2010). Furthermore, it was estimated that 18 million people suffered schistosome-related bladder wall pathology and 10 million suffered hydronephrosis.

Epidemiological studies in endemic human populations have shown that schistosome prevalence and intensity levels rise to peak in childhood (around ages 9–14) (2) and decline thereafter, so that in any endemic population, children carry the heaviest infection levels while adults carry little or no infection. This pattern has been taken to reflect the development of immune-meditated resistance to infection/re-infection (2,3) and suggests that protective immune responses develop slowly as a result of cumulative exposure to parasite antigens (4). Early serum transfer studies in the mouse model (5–8) as well as human immuno-epidemiological studies have shown that schistosome-specific antibody responses are associated with protection to infection and re-infection (9–13). Despite the demonstration that antibody-mediated responses can protect against schistosome infection in experimental models, current human schistosome vaccine research based on antibody-mediated protection has stalled with the failure of many of the vaccine candidate antigens to enter Phase III clinical trials (14). Limitations in our current understanding of the development of protective anti-schistosome responses against specific antigenic proteins may be contributing to the slow development of effective anti-schistosome vaccines. Previous studies characterizing immune responses to schistosome proteins have relied on recombinant proteins expressed in bacterial systems (15–17). However, there are restrictions associated with this approach; for example, it cannot detect immunogenic epitopes arising from post-translational modifications. Recent developments in two-dimensional gel electrophoresis and proteomics have the potential to overcome some of these restrictions because crude parasite antigen preparations can be separated into individual protein spots that can then be identified by mass spectrometry analysis and matched to newly available genomic databases.

The nature of the anti-schistosome antibody response to crude antigen preparations has been studied in terms of the different antibody isotypes involved and their relationship to each other, to host age and to schistosome infection level by ourselves and others (for example, see (4,11,18–21). Relatively few individual target antigens have been analysed in the context of selective antibody isotype recognition (22). IgA, IgE, IgG1 and IgG4 are of particular interest as they have been implicated in the development of protective human antischistosome immunity. Hagan *et al.* demonstrated a significant relationship between antischistosome IgE and IgG4 responses in *S. haematobium* infections. Furthermore, they reported a decrease in IgG4 accompanied by an increase in IgE with host age, which was associated with resistance to re-infection after treatment (10). We have previously reported that children exposed to *S. haematobium* produced a predominantly IgA response against egg and adult worm antigens which was gradually replaced by an IgG1 response in adulthood (4). These studies were based on immune responses to crude antigens containing a heterogeneous mixture of proteins and thus did not provide any information on the recognition patterns of individual antigens. Therefore, the antigenic source of variation in isotype-specific responses to *S. haematobium* is unknown. Differences may arise from the recognition of the same antigen by different antibody classes, from the recognition of different antigens or a combination of both. Identifying the individual antigens recognized by the different antibody isotypes in crude adult worm antigens would help resolve the cause(s) of some of these differences. In this study, we used proteomic approaches in combination with two-dimensional western blotting to determine which adult worm antigens are recognized by specific antibody isotypes/subclasses from schistosome-infected/exposed individuals. Thus, we identify the isotype-specific recognition patterns of individual parasite proteins within the crude adult *S. haematobium* proteome for the first time. We identified antigens recognized by IgA, IgE, IgG1 and IgG4, focussing our attention on WHO vaccine candidate antigens (23). Characterizing the antigens recognized by each antibody class will both improve our understanding of naturally acquired schistosome-specific immunity and inform vaccine development where the aim may be to stimulate an isotype-specific antibody response to a single recombinant antigen rather than a heterogeneous mixture of peptides. In this study, we determined the reactivity of sera from 7- to 18-year-olds, the age group where infection levels are changing from rising, peaking and declining in this population (24,25) to capture the range of immune reactivities as the dynamics of infection change. The analyses were conducted on pretreatment sera to study natural immune reactivities before enhancement of antibody reactivity with antihelminthic treatment, which we have previously demonstrated in a different subgroup from the same population (26).

## Materials and Methods

### Study area

The study was conducted in two rural villages in the Mashonaland East Province of Zimbabwe (31°30′E; 17°45′S) where *S. haematobium* is endemic. The study area is described in detail elsewhere (26). The study received ethical and institutional approval from the Medical Research Council of Zimbabwe and the University of Zimbabwe, respectively. Permission to conduct the work in this province was obtained from the Provincial Medical Director. The villages were selected because health surveys conducted regularly by the Ministry of Health and Child Welfare in the region showed little or no infection with other helminths and a low *S. mansoni* prevalence (<5%). The selected villages had not been included in the National Schistosome Control Programme and therefore had not received treatment for schistosomiasis or other helminth infections. The main activity in these villages is subsistence farming. Drinking water is collected from open wells while bathing and washing are conducted in two main rivers in the villages. Most families maintain a garden located near the river where water is collected for watering the crops, and the schools surveyed were all in close proximity to rivers.

### Study subjects

The study participants are described in detail elsewhere (26). Briefly, only permanent inhabitants of the villages who had never been treated for any helminth infection were eligible for inclusion in the study. Informed consent was obtained from all participants prior to enrolment into the study. Following explanation of the project aims and procedures to the community, school children and their teachers, an initial parasitology (stool and urine samples) and serology (blood sample) survey was conducted amongst all compliant participants. A questionnaire survey confirmed that on average, all participants frequented water contact sites at least four times per week and that frequency of water contacts at the various sites was not significantly different within the age range included in this study (data not shown).

Parasitology samples (at least two urine and two stool samples collected on 3 consecutive days) and 20 mL of venous blood were collected from each participant. Stool samples were processed following the Kato-Katz procedure (27) to detect *S. mansoni* eggs and other intestinal helminths, while the urine filtration method (28) was used to detect *S. haematobium* eggs in urine samples. After collection of the samples, all participants were offered treatment with the recommended dose of praziquantel, 40 mg/kg body weight. To be included in the cohort, participants had to meet all of the following criteria: (1) have provided at least two urine and two stool samples on consecutive days for the detection of helminth parasites; (2) have given a blood sample for serological assays; (3) be negative for intestinal helminths and *S. mansoni.* In practice, all people meeting criteria 1 and 2 were egg negative for intestinal helminths and *S. mansoni*, and so no participants were excluded on this third criterion. A total of 215 people (110 men and 105 women) aged from 5 to 18 years met all criteria. Serum samples obtained from 20 mL of venous blood from each participant were frozen and stored in duplicate at −20°C in the field and transferred to a –80 °C freezer in the laboratory. One complete set of the samples was subsequently transported frozen from Zimbabwe to the United Kingdom, stored at −80°C and defrosted for the first time for use in this study. A serum pool for use as a positive control in the western blot assays was made using equal volumes (50 μL) of sera from each of the 215 participants irrespective of their schistosome infection status and frozen in aliquots that were defrosted only once for the immunoassays.

### Parasite antigens

Adult worm antigens were used in this study as previous studies have shown that adult *S. haematobium* parasites suffer immune attrition and are targeted by host antibodies (29). Soluble *S. haematobium* adult worm antigens (SWAP) were obtained freeze-dried, from the Theodor Bilharz Institute in Egypt, and reconstituted as previously described (26). These were used in the subsequent gel electrophoresis and immunoassays.

### Antibody enzyme-linked immunosorbent assays

Circulating levels of IgA, IgE, IgG1 and IgG4 directed against the *S. haematobium* adult worm antigens were detected by indirect enzyme-linked immunosorbent assays (ELISA) as previously described (4) using 5 μg/mL of antigen to coat plates, sera diluted at 1 : 50 and the secondary horseradish peroxidase–conjugated secondary antibodies IgE (Sigma A9667, Sigma Aldrich, Gillingham, UK), IgG1(The Binding Site, AP006) and IgG4 (Ap009), all at 1 : 1000 dilution, and IgA (P0216; Dako, Cambridgeshire, UK) which was diluted at 1 : 2000. Negative control sera from five European volunteers who have never travelled to tropical countries and therefore presumed not previously exposed to schistosomiasis were also included in the assays to generate reactivity cut-off points.

### Gel electrophoresis

The soluble adult worm antigen preparation was separated into constituent proteins by two-dimensional gel electrophoresis on 13-cm gels as previously described (26) to generate a reference gel used for identifying proteins (200 μg of SWAP used) and the second (100 μg of SWAP used) for western blotting to determine which antigens were recognized by the sera following a previously established protocol (4).

### Determining sera and secondary antibody dilutions for western blotting

To minimize differences in sensitivity and specificity of the different assays arising from technical differences, assays were optimized by titration assays. A pool of sera from all of the Zimbabwean participants was used as a positive control, while a pool of sera from five European volunteers (same as the ones used in the ELISA assays above) was used as a negative control. The dilutions used for the sera and secondary antibodies (IgA, IgE, IgG1 and IgG4) were determined by a combination of enzyme-linked immunosorbent assay (ELISA) titration assays and trial western blots. First, ELISAs were conducted (varying both secondary antibody (1 : 500–1 : 2000) and sera dilutions (1 : 10–1 : 11280; see example in Figure S1) with 5 μg/mL of adult worm antigen. This informed the subsequent trial western blots conducted using sera diluted at 1 : 200 or 1 : 400 to allow enough sample for all replicate assays and to test a range of potential secondary antibody dilutions (1 : 1000, 1 : 2000, 1 : 4000) on a blot of 100 μg of two-dimensional electrophoresis-separated SWAP. From these data, we determined dilutions that gave the most consistent replicates and optimal sensitivity and specificity for each antibody isotype. Higher dilutions of secondary antibodies resulted in the detection of fewer antigenic spots (see Figure S2), while reducing the secondary antibody dilution factor from 1 : 1000 (IgE, IgG1, IgG4) or 1 : 2000 (IgA) resulted in higher background rather than an increase in the number of spots detected. Furthermore, sera and secondary antibody dilutions used were consistent with those we have previously used in ELISA (24,30) and western blot assays (16), enabling our results to be related to previous studies. The finalized protocol is described elsewhere.

### Western blotting

Western blotting was used to determine the proteins reactive with the isotypes IgE, IgG4, IgA and IgG1 in sera from the participants. To achieve this, proteins were transferred from the gel onto nitrocellulose membrane as previously described (26). Blots were run in pairs (one gel/membrane) to probe for two antibody isotypes at a time in parallel assays. To verify efficient transfer of SWAP proteins, membranes were stained with Ponceau S solution (Sigma) and then blocked for 1 h at room temperature in TBS blocking buffer [Pierce, (Thermo Scientific), Surrey, UK] with 0.05% Tween 20. This was followed by 2 × 10 min washes with TBS/0.05% Tween 20/0.5% Triton-X 100 (TBS/TT) (used for all washes). The pool of positive control sera (diluted at 1 : 200 in TBS blocking buffer with 0.02% Tween 20) was added to each of the membranes and incubated overnight at 4°C followed by 3 × 10 min washes. Horseradish peroxidise–conjugated secondary antibodies diluted in TBS blocking buffer/0.05% Tween 20 were then added to respective membranes diluted at 1 : 1000 for IgE and 1 : 2000 for IgA. Membranes were subsequently incubated at room temperature for 1 h, followed by 4 × 10 min washes in TBS/TT and 1 × 10 min washes in TBS alone. The proteins were visualized using the chemiluminescence product ECL Plus (GE Healthcare, Buckinghamshire, UK) according to the manufacturer’s instructions. Films exposed to the blots for 5 s were developed and spots matched to the Coomassie blue–stained reference gel using the image analysis software Image master. Following visualization, the membranes were stripped of the ECL reagent, secondary antibody and sera following the manufacturer’s protocol. The same membranes were then re-probed using the same pool of sera. The IgE membrane was re-probed with IgG4 diluted at 1 : 1000, while the IgA membrane was re-probed with IgG1 diluted at 1 : 1000. A previous assay showed that the stripping procedure removed all proteins not directly bound to the nitrocellulose membrane as indicated by the lack of ECL reactivity with a stripped membrane. This procedure did not remove any of the parasite proteins as evidenced by probing the same membrane with three serum samples successively, i.e. sera from the endemic population, followed by negative control sera (a pool from five Europeans who had never travelled to tropical regions) and then by the same sera from the endemic population. An example of a membrane blotted with negative control sera and the IgG4-specific secondary antibody is shown in Figure S3. Gel electrophoresis and western blotting were repeated twice using the secondary antibodies in different order on the same membrane, i.e. IgG1 followed by IgA and IgG4 followed by IgE to confirm the recognition patterns obtained.

### Image analysis and mass spectrometry

Images from the western blots were electronically scanned for image analysis. Spots on these blots were detected by pixel analysis and matched across the different blots for each isotype using the 2-D gel image analysis software ImageMaster from GE Healthcare. All electronic spot identifications and predicted matches were manually verified via independent visualization by two researchers. Spots on the Coomassie blue–stained gel, which matched to those on the western blots, were excised, digested with trypsin and analysed by mass spectrometry analysis as previously described (26). Data thus obtained were submitted to an MSMS ion search via the Mascot search engine (Matrix Science), searching both locally established databases for *S. mansoni* EST sequence and the current nonredundant NCBI database.

### Data analysis

The ratios of IgG1/IgA and IgE/IgG4 were calculated for each individual from ELISA data for each individual. Partial correlations were conducted to determine the association between the ratios and schistosome infection intensity allowing for residential village, age and sex. Significance level for the statistics tests was set at *P* < 0.05. For all analyses of antigenic spots, if the recognition intensity of the spot after subtraction of the background intensity was 0 pixels, then the spot was designated ‘not recognized’ by the isotype, and if it was greater than 0, then the antigenic spot was designated as ‘recognized’. To allow comparisons of the intensity of antigen recognition by the different isotypes, the detected spots were categorized by their recognition intensity into five groups; group 1 comprised all antigens not recognized by the pooled sera and groups 2–5 were based on the interquartile ranges for each isotype using the mean intensity from the three replicate blots per isotype. The interquartile ranges were calculated from the antigens using a spot detected from pixel analysis and manual verification. These ranges were calculated for each isotype separately so that the spot intensities were standardized within the gel. The intensity categories rather than absolute values could then be compared across the different gels. This approach was used as it does not require any spots to be recognized equally by the four isotypes for calibrating recognition intensity across all isotypes.

## Results

### Levels of IgA, IgE, IgG1 and IgG4 detected in individuals

**Figure 1:**
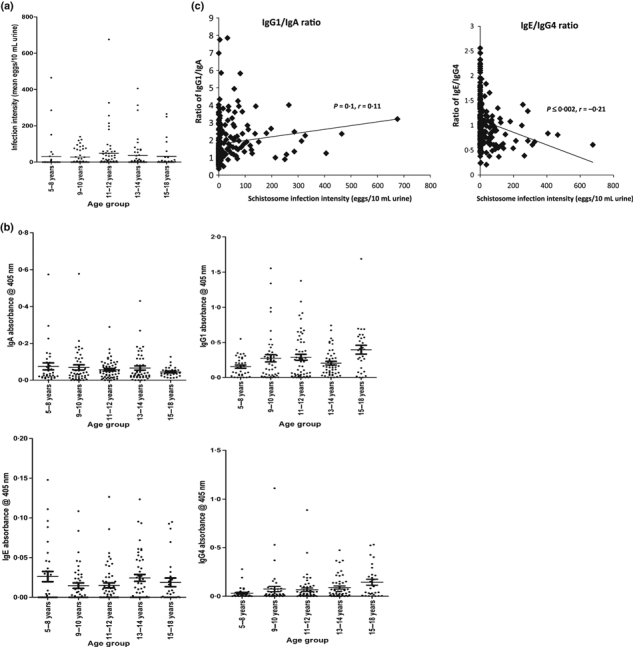
Description of the individual study participants partitioned by age group. (a) Infection intensity for each individual calculated as a mean of eggs/10 mL of urine from at least two urine samples collected on consecutive days. (b) Antibody levels measured as optical densities for each individual (c) The relationship between the ratios of IgG1/IgA and IgE/IgG4 calculated from the values in (1B) above and schistosome infection intensity for each individual participant. These show a significant negative association between the ratio IgE/IgG4 and infection intensity and no significant association between the IgG1/IgA ratio and infection intensity.

The prevalence of *S. haematobium* infection in the participants was 57%, and the corresponding mean infection intensity was 36 eggs/10 mL urine (SE of the mean = 5.7), with individual egg counts ranging from 0 to 676 eggs/10 mL urine. Individual infection intensity followed the typical age infection profile for schistosome infections, rising with age to peak in childhood and then declining thereafter as shown in Figure 1a. The levels of the four antibody isotypes IgA, IgE, IgG1 and IgG4 directed against adult worm antigen were measured for each individual who was included in the serum pool. A participant was considered reactive for an isotype if their titre was above the cut-off point of mean +2 standard deviations of the negative control sera. Based on this cut-off point, the least prevalent schistosome-specific isotype was IgA being detected in 31% of the participants and then IgE detected in 50% of the participants. IgG1 was detected in 58% of the participants, while IgG4 was the most prevalent response, detected in 74% of the participants. Levels of the isotypes were plotted by age groups in Figure 1b.

Earlier studies have shown that higher ratios of IgG1/IgA and IgE/IgG4 are associated with lower infection levels (10,31). Therefore, to determine whether the relationship between *S. haematobium* infection and antibody responses in this study was consistent with those reported from other earlier studies, the ratios of IgG1/IgA and IgE/IgG4 were calculated and plotted against schistosome infection intensities. The ratio of IgE/IgG4 showed a significant inverse relationship with schistosome infection intensity (*r* = −0.21, *P* = 0.002, df = 210), so that people with high values for these ratios carried little or no schistosome infection (Figure 1c). The IgA/IgG1 ratio did not show a significant association with infection intensity (*r* = 0.11, *P* = 0.10, df = 210). The subsequent western blotting analyses were performed to determine whether these different isotypes recognized the same individual native antigens.

### Antigens recognized by the different isotypes

The potential repertoire of schistosome antigens recognized on two-dimensional blots of adult parasite extract was first defined with a highly reactive positive post-treatment serum pool made from a subgroup of this population previously used to investigate the effects of praziquantel treatment on schistosome-specific responses (26), which was probed with a secondary antibody combination reactive to IgA/IgG/IgM. This serum pool reacted with a total of 71 spots compared to negative nonendemic sera that reacted with none of the protein spots. The 71 proteins were identified and characterized in our previous study (26) which gives their predicted molecular weights, isoelectric point (p*I*), Mascot output statistic and accession numbers from NCBI.

**Figure 2:**
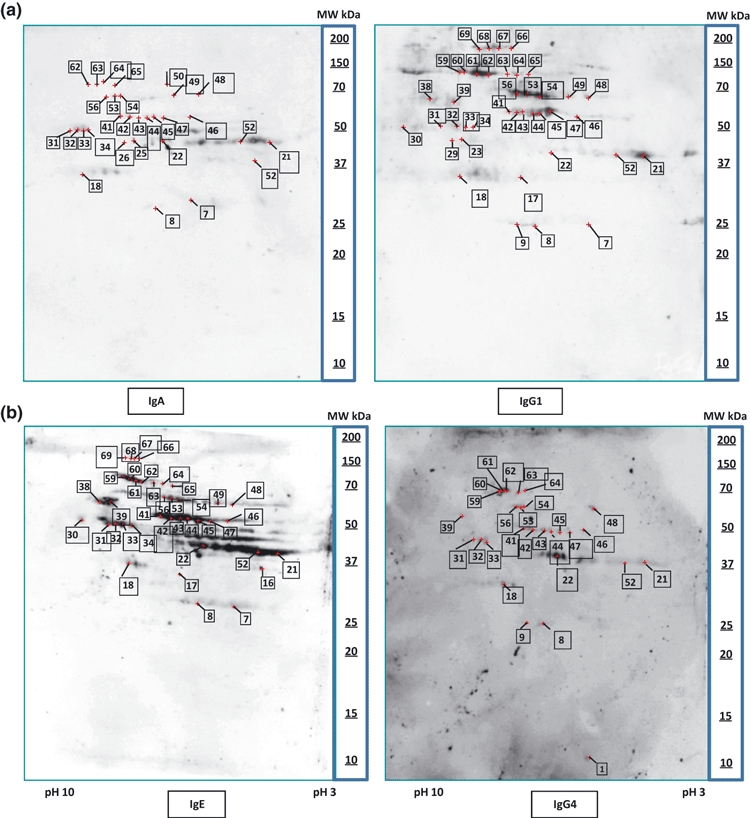
Antigen recognition patterns of the serum pool compared by two-dimensional western blotting. (a) IgA vs. IgG1. (b) IgE vs. IgG4.

Compared to the IgA/IgG/IgM positive control, no single isotype in the serum pool recognized all of the 71 antigenic spots. The differences in recognition of the antigenic spots between the isotypes were both qualitative and quantitative. IgG1 recognized the largest number of spots (*n* = 45) followed by IgA and IgE which both recognized 39 spots, with IgG4 recognizing the fewest antigenic spots (*n* = 35) as shown in Figure 2 and Table 1. The most strongly recognized antigens by all subclasses were the two isoforms of glyceraldehyde-3-phosphate, some isoforms of heat shock protein (HSP) 70 as well as some proteins still to be identified (spots 60 and 61). The antigenic spots 2 (protein yet to be indentified), 6 (triose-phosphate isomerase) and 15 (yet to be identified) were strongly recognized by IgG1 but were not detected by any of the other isotypes. There were two isoforms of fructose-1-6-bis-phosphate aldolase, one reactive with IgA (antigenic spot 25 in Figure 2a) and one reactive with IgG1 (antigenic spot 23 in Figure 2a). Some antigens were not recognized at all byany of the 4 isotypes tested; these included the muscle proteins myosin (heavy and light chain), tropomyosin and paramyosin, HSP 60 and some isoforms of enolase and immunophilin. Some antigenic spots such as those between spots 22 and 52 in Figure 2 were strongly recognized by the sera but were in such low abundance in the Coomassie reference gel that they could not be isolated and identified by mass spectrometry as reported in previous studies (26,32).

**Table 1:** The table gives identities of the antigenic spots in Figure 3, relating the spot number to its protein identity from the peptide searches and recognition intensity by each of the isotypes

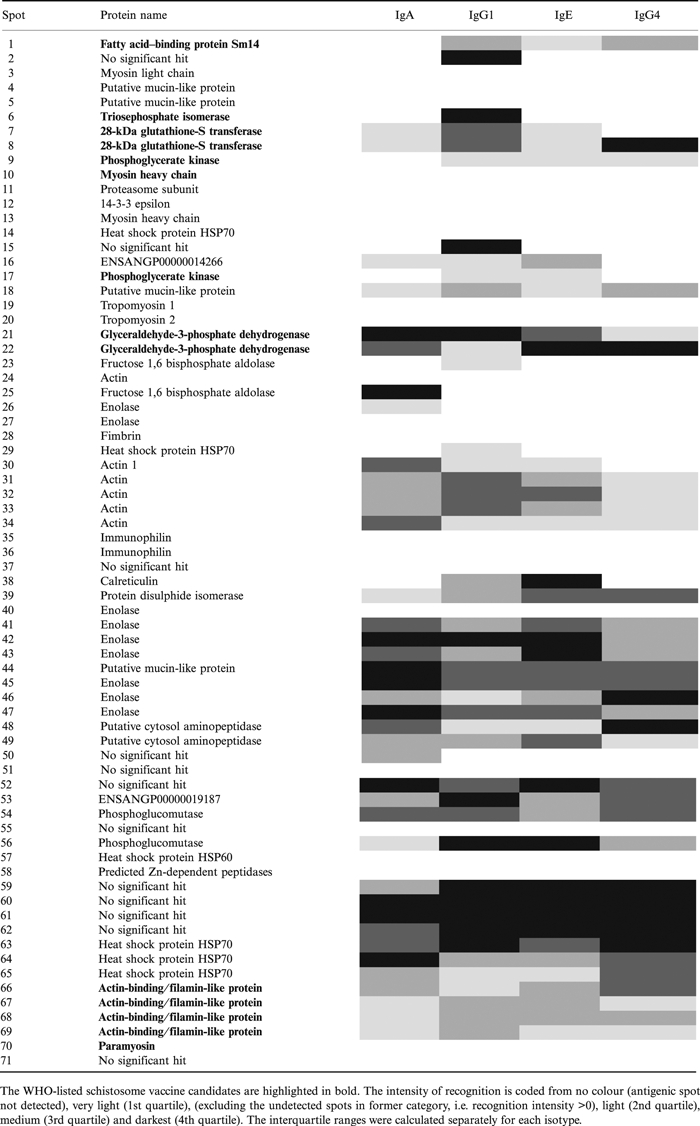

**Figure 3:**
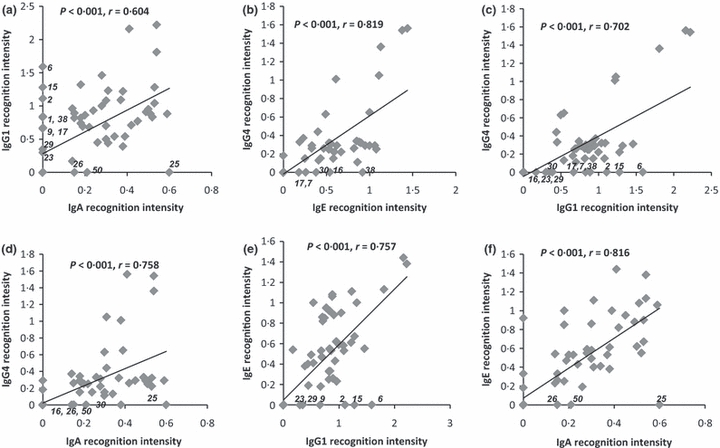
Comparison of recognition intensity for each antigenic spot by the different isotypes showing a significant positive correlation between all 4 isotypes. Italicized numbers refer to the spot identity of antigenic spots preferentially recognized by one of the two isotypes, and their protein identities are given in Table 1. (a) IgG1 vs. IgA. (b) IgG4 vs. IgE. (c) IgG4 vs. IgG1. (d) IgG4 vs. IgA. (e) IgE vs. IgG1. (f) IgE vs. IgA.

To quantify the relationships between protein spot recognition by different isotypes, the intensity of recognition of each antigenic spot by each isotype was categorized by interquartile range (to reduce the effects of differences arising because of intra-isotype variation in detection thresholds) as shown in Table 1. The recognition intensity of the spots was positively correlated so that for most spots, those intensely recognized by IgA were also intensity recognized by IgG1 (Figure 3a). Thirty-six of the 39 antigenic spots recognized by IgA were also recognized by IgG1 (Table 1). However, IgG1 recognized an additional nine spots indicated in Figure 3a (numbered spots on the *y* axis). These resolved to the antigens fatty acid–binding protein (Sm14), phosphoglycerate kinase, triose-phosphate isomerase, an isoform of fructose 1-6-bis-phosphate aldolase, HSP 70, calreticulin, and two proteins yet to be identified (spots 2 and 15). Conversely, IgA recognized three antigenic spots not recognized by IgG1 (numbered spots on the *x*-axis of Figure 3a). These were an isoform of enolase, an isoform of fructose 1-6-bis-phosphate aldolase, and a protein (spot 50) yet to be identified. These were also not recognized by IgG4 (Figure 3d) or IgE (Figure 3f).

Similar patterns of correlations between isotypes, but with some antigens being exclusively or largely recognized by only one isotype, were seen in all other pairwise comparisons (Figure 3). IgE recognized all 34 antigenic spots recognized by IgG4 as shown in Table 1 and Figure 3b. In addition, it also recognized an isoform of 28-kDa glutathione-S-transferase, phosphoglycerate kinase, actin 1, calreticulin and one protein (spot 16) still to be identified.

Of all recognized antigens, only triose-phosphate isomerase (spot 6 in Figure 2 and Table 1), isoforms of fructose 1-6-bis-phosphate aldolase (spots 23 and 25), enolase (spot 26), HSP70 (spot 29) and spots 2 and 15 which are proteins still to be identified were recognized by a single isotype. The isoforms of fructose-1-6-bis-phosphate aldolase were reactive with either IgA (antigenic spot 25 in Figure 2a) or IgG1 (antigenic spot 23 in Figure 2a) but not with both. IgG1 recognized all of the antigenic spots recognized by IgG4 (Table 1, Figure 3c) and IgE (Table 1, Figure 3e).

Of the current World Health Organisation’s 10 schistosome vaccine candidates, only three were recognized by all of the isotypes assayed. These were an isoform of 28-kDa glutathione-S-transferase (spot 8), both isoforms of glyceladeyde-3-phosphate (spots 21 and 22) and several isoforms of actin-binding/filamin-like protein (spots 66–69). The vaccine candidate paramyosin was not recognized by any of the antibodies assayed.

## Discussion

Passive transfer studies in animal models show that antibodies can protect against schistosomiasis (5–8,33). In parallel, human immuno-epidemiology studies have shown that some antibody classes are associated with low levels of infection in endemic populations as well as low re-infection rates following treatment with antihelminthic drugs (10,12,13,34–36). However, all previous studies to date have focused on describing antibody responses either to individual recombinant antigens or to uncharacterized ‘whole worm’ antigen preparations. We have previously defined native schistosome adult proteins recognized by the total IgG fraction of human serum from samples collected from this same population (26). In this study, we compare the reactivity of different isotypes to the soluble component of adult worm crude antigen, separated into its constituent defined antigens, to answer two questions. First, which antigens are recognized by the different antibody iostypes IgA, IgE, IgG1 and IgG4? Second, do different isotypes recognize the same antigens? It is possible that observed differences in recognition intensity reflect not only the biological (functional) differences between the isotypes but also differences in the sensitivity and specificity of the isotypes, so that some antigens would be below the detection limit of the assays. However, we have carefully calibrated the dilutions of sera and secondary antibodies to enable optimal comparison and minimize this possibility. We have used three replicates of each blot to verify the patterns and ratios of recognition intensity of each protein spot that we observed for each of the four isotypes. Thus, we have shown that some antigens from within the adult *S. haematobium* proteome are preferentially recognized by certain isotypes. For example, one isoform of the antigen fructose-1-bis-phosphate aldolase was intensely recognized by IgA but not detected by IgE, IgG1 or IgG4. Furthermore, comparing recognition intensity between isotypes using quartiles calculated within each subclass (Table 1), rather than absolute values (Figure 3), corrected for some intra-isotype variation in sensitivity. The study was designed to extend our understanding of the individual antigens detected in immunoassays routinely conducted by ELISA using crude antigens. In these former assays, protocols are designed to optimize sensitivity and specificity as carried out here to reduce the effects of differences in detection thresholds of the different isotypes.

The four isotypes we investigated detected a total of 48 antigenic spots of a possible 71 spots detected with total IgG antibodies previously described by screening adult worm antigens with post-treatment sera (26). The sera used in this current study were from 7- to 18-year-olds before enhancement of antibody reactivity with antihelminthic treatment (26). Our previous study involved older people, i.e. up to 42 years old, and included the reactivities of the additional IgG subclasses IgG2, IgG3 which are reactive against some of the schistosome vaccine candidates (23). Therefore, it is not surprising that the number of spots recognized by the IgG subclasses (IgG1 and IgG4) in this study would be fewer than in previous studies. Comparing the number of antigenic spots detected and the prevalence of the response in the study population, it is interesting that while IgG4 was the most prevalent response in the study population (present in 74% of the population), this isotype detected the least number of antigenic spots, suggesting that there are few dominant antigens stimulating the IgG4 response. Conversely, IgG1 detected in 58% of the participants, recognized the largest number of spots, suggesting that the IgG1 response is directed against a larger repertoire of antigens than the other isotypes.

We have previously shown a dichotomous association between IgA and IgG1 responses directed against adult worm antigens in a different Zimbabwean population (13), and the spot identification in Figure 3a shows that there are some antigens recognized either by IgG1 or by IgA but not by both antibodies. An inverse relationship has also been reported in a case of idiopathic arthritis where patients deficient in IgG1 had high titres of IgA (37), suggesting that the expression of these two subclasses is differentially regulated. IgG1 recognized the largest number of antigenic spots: 45 including 8 of the 10 of the WHO’s schistosome vaccine candidates. Furthermore, in this study, IgG1 levels rose with age to peak in the oldest age group with the least infection, suggesting that the antibody is associated with protection against infection. This is consistent with our previous studies showing an association between antiworm IgG1 responses and protection to *S. haematobium* infection (13). IgA recognized fewer antigenic spots than IgG1, reacting with only 3 of the 10 schistosome vaccine candidates including the leading vaccine candidate 28-kDa glutathione-S-transferase.

The ratio of IgE to IgG4 levels has previously been associated with protection to re-infection by *S. haematobium* infection (10), and the association of a high IgE/IgG4 ratio with little or no infection in our present study is consistent with these findings. The inverse relationship between IgG4 and IgE has been used to suggest that IgG4 antibodies might block IgE effector functions by competitively binding to epitopes recognized by both isotypes and thereby inhibit IgE-dependent cell cytotoxicity mediated by monocytes, eosinophils or platelets (11,38). The IgG4 subclass is upregulated in association with anti-inflammatory factors [for example, IL-10 (39)] and its own anti-inflammatory characteristics are thought to help the immune system to dampen inappropriate inflammatory reactions (40). This would only be possible if the two isotypes recognized the same antigens. In this study, IgG4 bound to the fewest antigenic spots, while IgE recognized all those recognized by IgG4. IgE reacted with seven of the 10 vaccine candidates, five of which were also recognized by IgG4. The recognition intensities (for each antigenic spot representing both affinity and avidity) of these two antibodies were the most strongly correlated in the study, suggesting a tightly regulated association between them. Earlier studies in filariasis using western blotting of one-dimensional gels indicated that the level of cross-reactivity between IgE and IgG4 differed with the level of pathology (41), which suggests that during the course of infection, there is dissociation in antigen recognition patterns between the two antibodies. How this dissociation occurs is unclear but may be related to the order of antibody class switching where IgG4-secreting B cells can switch to IgE but not the converse (42) and/or a threshold being reached in antigen levels (32).

The antigens recognized by IgE but not IgG4, and by IgG1 but not IgA, are of interest as they may be associated with the development of resistance to schistosomiasis; this is supported by the fact that most of these antigens are already known vaccine candidates (23). Studies are now underway to identify the proteins that were not serologically reactive, particularly those that are abundant in the soluble proteome which might play an important role in immune evasion and/or modulation of the host immune response (43). Similarly, there is a need to identify the intensely recognized proteins that occurred at insufficient concentrations in the adult worm proteome to be identified by mass spectrometry, as these may indicate highly immunogenic vaccine candidates as yet undiscovered.

Overall, our study has indicated that although the majority of the antigens are recognized by all of the four antibody isotypes tested, there may be restriction in antigen recognition for some antigens, for example prevalent isotypes such as IgG4 appear to react to relatively few of the major adult worm antigens. This may partly explain differences observed in population level isotype dynamics in human schistosomiasis. Furthermore, there are differences in the recognition of isoforms of some antigens such as the leading vaccine candidate, 28-kDa glutathione-S-transferase and glyceraldehyde-3-phosphate dehydrogenase. These isoform-specific differences are an important consideration for vaccine development, where recombinant vaccines lack post-translational modifications and therefore epitopes crucial for induction of the relevant isotype-specific antibody responses. Studies on antigen recognition patterns by different antibodies present in sera from individuals or partitioning the study population by age and infection status as we previously reported for total IgG will be valuable for identifying further vaccine candidates (32).

## SUPPORTING INFORMATION

**Figure S1.** Enzyme-linked immunosorbent titration assays to optimize the sensitivity and specificity of the isotypes used in the assay using adult worm antigen at 5 μg/mL.

**Figure S2.** Western blot assays used to optimize the sensitivity and specificity of the isotypes used.

**Figure S3.** Results of the western blot assays using the negative control sera (pooled from five European donors who have never travelled to tropical countries) diluted at 1 : 200 and IgG4 secondary antibody diluted at 1 : 1000 showing no reactivity of the negative control sera with schistosome adult women antigens.
