# Supplementary material for: Differential recognition patterns of Schistosoma haematobium adult worm antigens by the human antibodies IgA, IgE, IgG1 and IgG4
Source: Parasite Immunol. 2011 Mar;33(3):181–92. doi: 10.1111/j.1365-3024.2010.01270.x (PMC3084999; doi:10.1111/j.1365-3024.2010.01270.x)
Supplement: Supplementary file 1 [file pim0033-0181-SD1.ppt]

## Slide 1
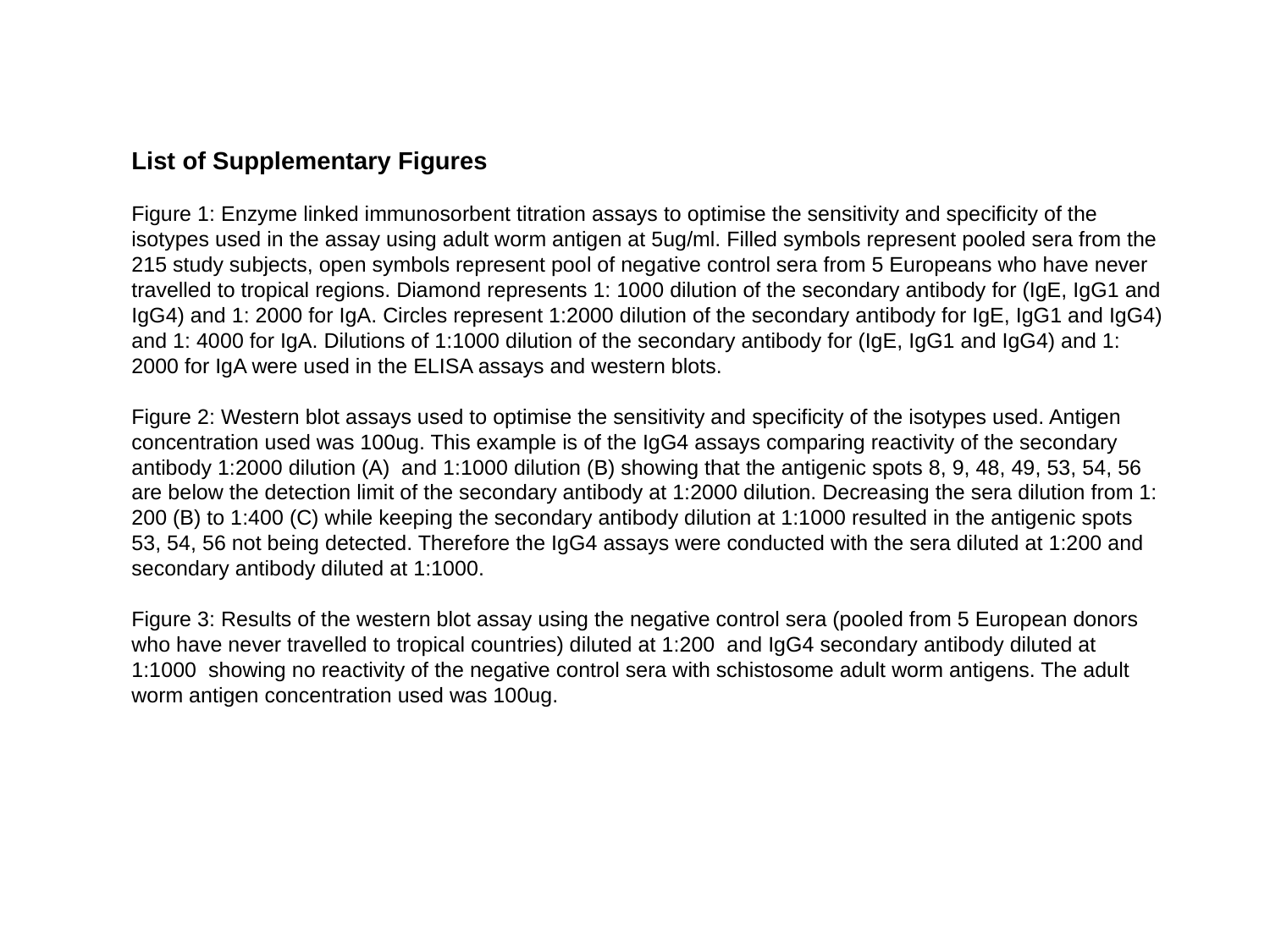

List of Supplementary Figures
Figure 1: Enzyme linked immunosorbent titration assays to optimise the sensitivity and specificity of the isotypes used in the assay using adult worm antigen at 5ug/ml. Filled symbols represent pooled sera from the 215 study subjects, open symbols represent pool of negative control sera from 5 Europeans who have never travelled to tropical regions. Diamond represents 1: 1000 dilution of the secondary antibody for (IgE, IgG1 and IgG4) and 1: 2000 for IgA. Circles represent 1:2000 dilution of the secondary antibody for IgE, IgG1 and IgG4) and 1: 4000 for IgA. Dilutions of 1:1000 dilution of the secondary antibody for (IgE, IgG1 and IgG4) and 1: 2000 for IgA were used in the ELISA assays and western blots.
Figure 2: Western blot assays used to optimise the sensitivity and specificity of the isotypes used. Antigen concentration used was 100ug. This example is of the IgG4 assays comparing reactivity of the secondary antibody 1:2000 dilution (A) and 1:1000 dilution (B) showing that the antigenic spots 8, 9, 48, 49, 53, 54, 56 are below the detection limit of the secondary antibody at 1:2000 dilution. Decreasing the sera dilution from 1: 200 (B) to 1:400 (C) while keeping the secondary antibody dilution at 1:1000 resulted in the antigenic spots 53, 54, 56 not being detected. Therefore the IgG4 assays were conducted with the sera diluted at 1:200 and secondary antibody diluted at 1:1000.
Figure 3: Results of the western blot assay using the negative control sera (pooled from 5 European donors who have never travelled to tropical countries) diluted at 1:200 and IgG4 secondary antibody diluted at 1:1000 showing no reactivity of the negative control sera with schistosome adult worm antigens. The adult worm antigen concentration used was 100ug.

## Slide 2
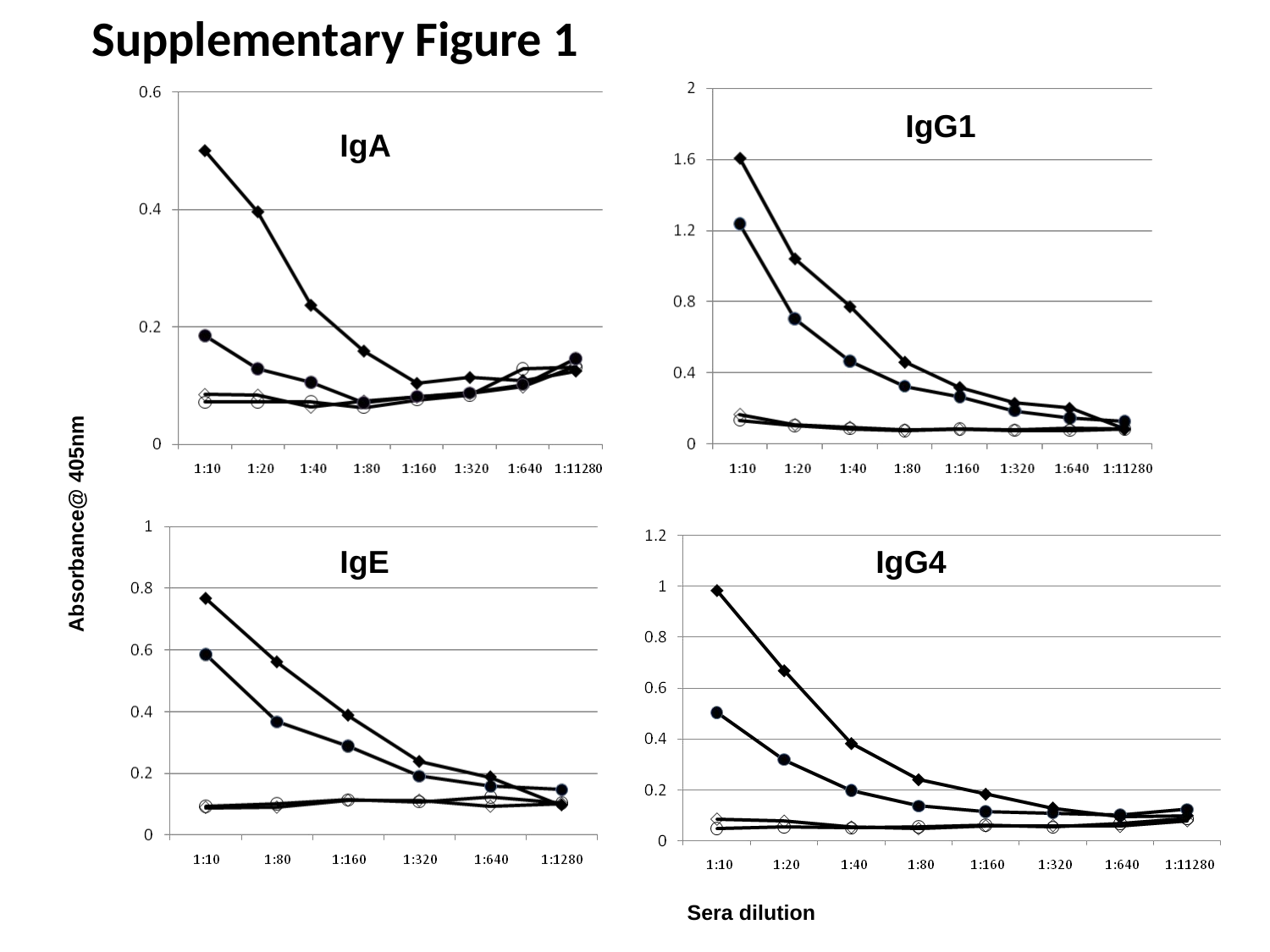

Supplementary Figure 1
IgG1
IgA
Absorbance@ 405nm
IgE
IgG4
Sera dilution

## Slide 3
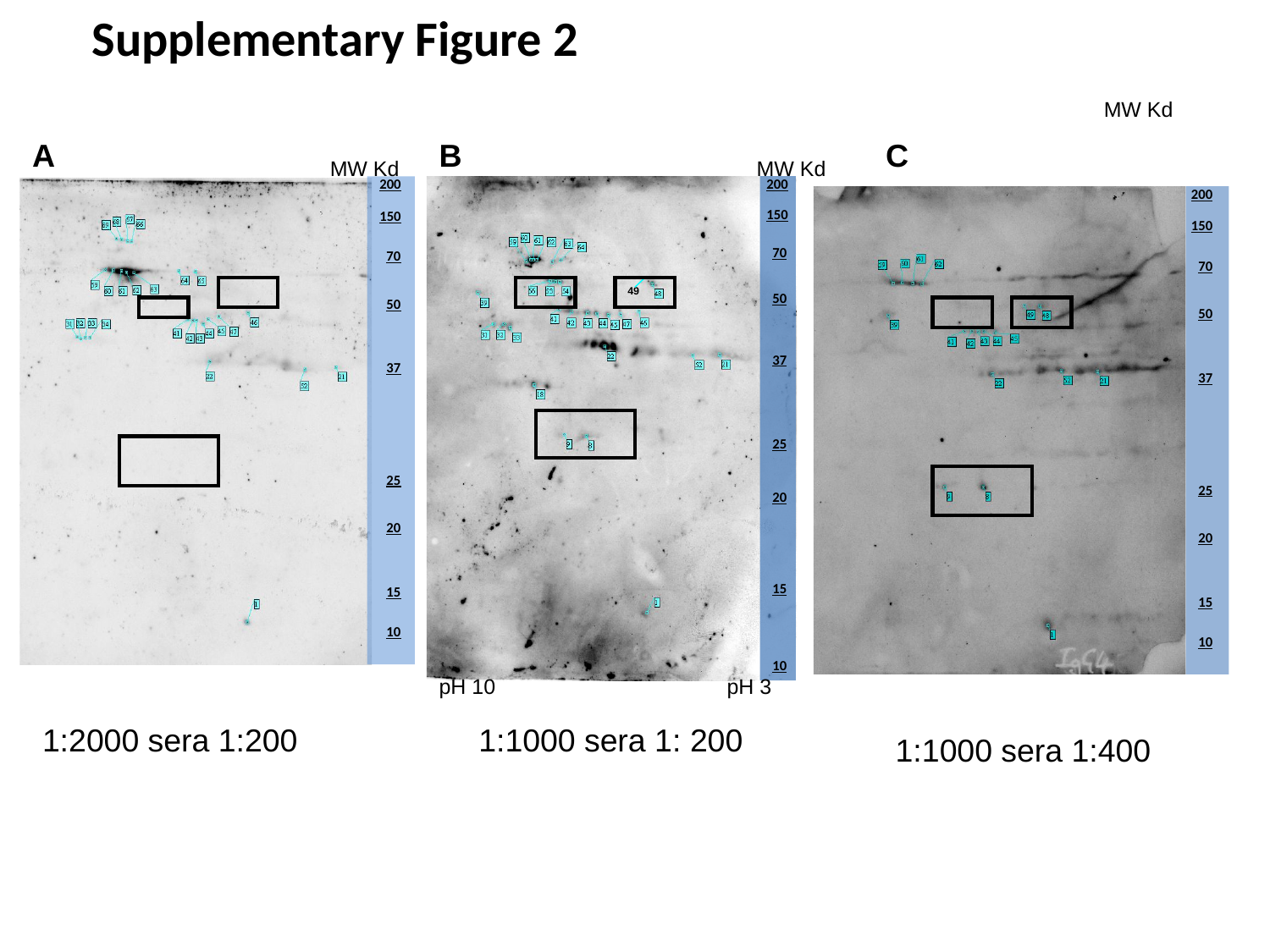

Supplementary Figure 2
MW Kd
A
B
C
MW Kd
MW Kd
200
150
70
50
37
25
20
15
10
200
150
70
50
37
25
20
15
10
200
150
70
50
37
25
20
15
10
49
pH 10
pH 3
1:1000 sera 1: 200
1:2000 sera 1:200
1:1000 sera 1:400

## Slide 4
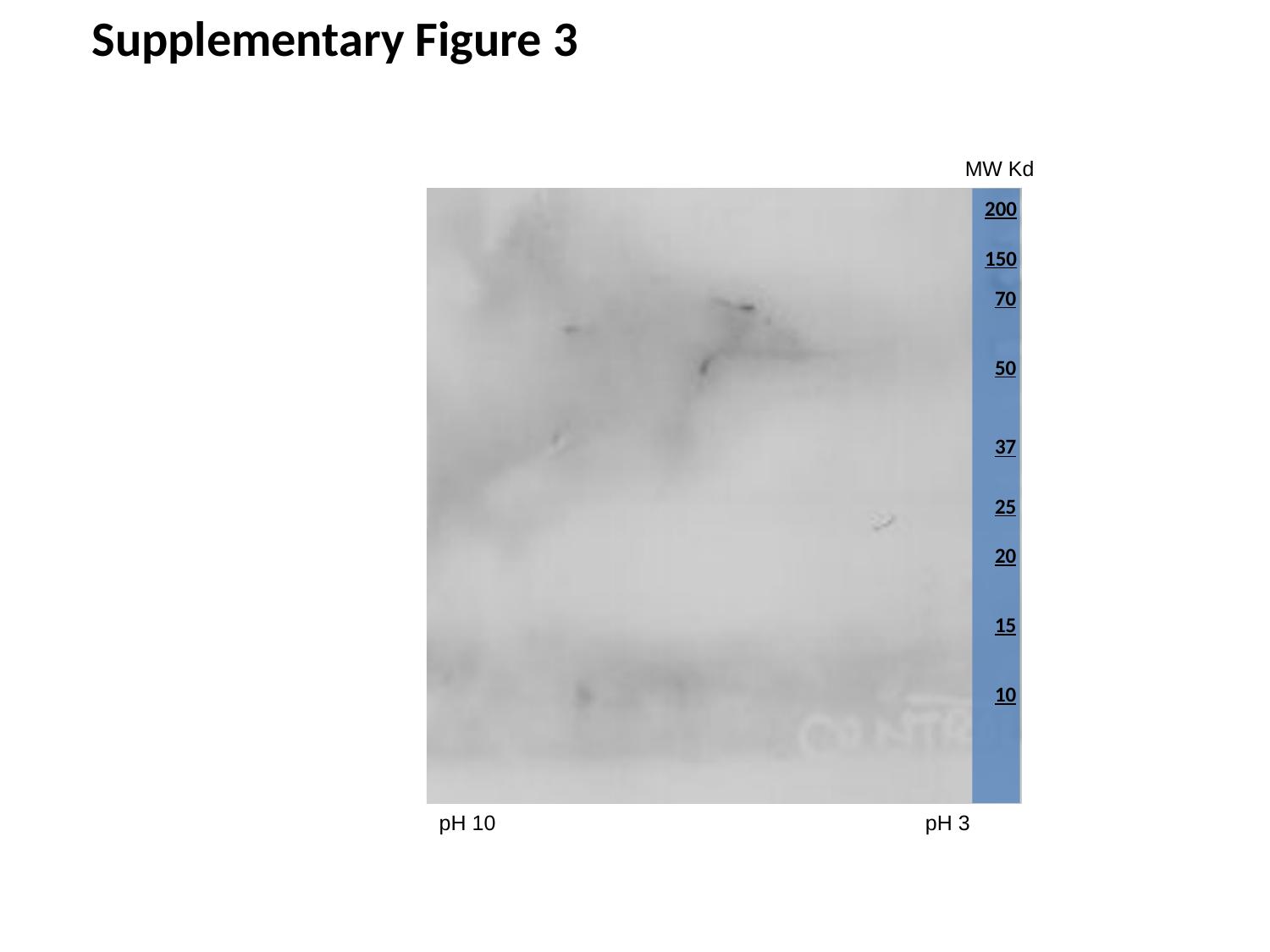

Supplementary Figure 3
MW Kd
200
150
70
50
37
25
20
15
10
pH 10
pH 3
